# Altered sense of agency in schizophrenia: the aberrant effect of cardiac interoceptive signals

**DOI:** 10.3389/fpsyt.2024.1441585

**Published:** 2024-09-25

**Authors:** Akihiro Koreki, Yuri Terasawa, Atsuo Nuruki, Hiroki Oi, Hugo Critchley, Mahinda Yogarajah, Mitsumoto Onaya

**Affiliations:** ^1^ Department of Psychiatry, NHO Shimofusa Psychiatric Medical Center, Chiba, Japan; ^2^ Department of Psychiatry, NHO Chibahigashi Hospital, Chiba, Japan; ^3^ Department of Neuropsychiatry, Keio University School of Medicine, Tokyo, Japan; ^4^ Department of Psychology, Keio University, Tokyo, Japan; ^5^ Center for General Education, Institute for Comprehensive Education, Kagoshima University, Kagoshima, Japan; ^6^ Department of Clinical Neuroscience, Brighton and Sussex Medical School, Sussex University, Brighton, United Kingdom; ^7^ Department of Clinical & Experimental Epilepsy, University College London (UCL) Queen Square Institute of Neurology, London, United Kingdom

**Keywords:** brain, interoception, heart, schizophrenia, sense of agency, self

## Abstract

**Background:**

Schizophrenia (SZ) is characterized by abnormalities in self-representation, including a disturbed sense of agency (SoA). The continuous processing of sensory information concerning the internal state of the body (interoception) is argued to be fundamental to neural representations of the self. We, therefore, tested if aberrant interoception underpins disturbances in SoA in SZ, focusing on cardiac interoceptive signaling.

**Methods:**

Forty-two SZ and 29 non-clinical participants (healthy controls; HC) performed an intentional binding task to measure SoA during concurrent heartbeat recording. The effect of cardiac interoceptive signals on SoA was measured by the difference in intentional binding effect during systole and diastole. This measure was standardized based on the overall intentional binding effect to control for non-cardiac factors, and then compared between SZ and HC.

**Results:**

Our study revealed a significant difference between SZ and HC groups, with opposite effects of cardiac systole on SoA. Specifically, cardiac systole disrupted SoA in SZ, contrasting with the enhanced SoA in HC. Across the SZ group, the extent to which SoA was disrupted by cardiac systole correlated significantly with a clinical proxy for symptom instability, namely the number of hospital admissions for hallucinations and delusions. Furthermore, the disruption was particularly observed in patients with severe hallucinations.

**Conclusions:**

This study revealed a disturbance in the impact of cardiac interoceptive signals on an implicit index of SoA in schizophrenia. This supports the notion that pathophysiological disruption of the central integration of interoceptive information increases vulnerability to disturbances in self-representation and the associated expression of schizophrenic symptoms.

## 1 Introduction

The sense of self is a fundamental aspect of human conscious experience. Alterations in the sense of self (i.e. ‘self-disturbances’) are argued to be a core feature of schizophrenia (1). Two aspects of the minimal (biological) self are proposed: The sense of agency (SoA) and the sense of ownership (2). SoA is the sense that “I” am the one who is causing or generating an action and its effect, while the sense of ownership is the sense that a body is “mine”. Abnormalities in the sense of self, especially in the SoA, have been experimentally demonstrated in individuals with schizophrenia (SZ) (3–8). The sense of self emerges from the integration of different factors (3, 8), yet recent studies highlight a core role of interoception in shaping self-representations (9–16).

Interoception encompasses the signaling, central processing, and (unconscious and conscious) representation of information about the internal bodily state (17–20). Interoception is distinct from exteroception, which addresses external information about the environment, and proprioception, which concerns the position of the body in space. A hierarchical framework is proposed for the central representation of interoceptive information, from basic neural signaling with implicit representation that can nevertheless influence actions, cognitions, and perceptions to higher-level, consciously accessible, explicit representations of internal bodily sensations, such as the feeling of one’s heart beating or stomach churning. The representation of cardiovascular information is of particular importance since dynamic changes in the state of cardiovascular arousal (e.g. increases in heart rate and blood pressure) accompany action-orientated behaviors and emotions. Arterial baroreceptors located in the aortic arch and carotid sinus are the origin of a major route for cardiac interoception. These stretch receptors increase their firing when blood is ejected from the heart at ventricular systole, informing the brain about the timing and strength of each heartbeat and, hence, the state of cardiovascular arousal. These phasic signals, conveyed by the vagus and glossopharyngeal nerves, are first processed in the brainstem (for blood pressure regulation) and ascend the neuraxis to influence behavioral and psychological functions via subcortical and cortical representations.

Cardiac interoceptive signaling influences both emotion and self-representation (11, 14, 16, 18). Accurate detection and awareness of heartbeat sensations may shape emotional feelings and the experience of anxiety: As a result, much attention has been given to examining individual differences in conscious perceptual sensitivity to heartbeat sensations (as indexed by neuropsychological tasks, such as the heartbeat counting task and the heartbeat discrimination tasks) (20, 21). However, phasic cardiac signals also influence many mental processes implicitly, i.e. independently of conscious awareness and appraisal. In self-representation, the sense of agency (SoA) captures one’s subjective experience of controlling one’s actions and action consequences. This can be measured objectively and implicitly using an intentional binding task (8). Intentional binding is a perceptual phenomenon characterized by the subjective compression of the temporal interval between a voluntary action and its subsequent outcome (8). Intriguingly, intentional binding has been shown to be sensitive to cardiac interoceptive signals (14, 16): In (non-clinical) participants, the SoA inferred from enhanced intentional binding is greater during cardiac systole than diastole (14). Moreover, people with greater perceptual sensitivity to heartbeat signals (more accurate performance on a heartbeat discrimination task) show enhanced SoA, particularly at cardiac systole, relative to individuals with poorer interoceptive accuracy, while heart rate and heart rate variability were not relevant to this effect (16).

There are several potential mechanisms that might explain why better performance on behavioral cardiac interoceptive tasks may modulate the SoA (16). First, since the sense of ownership and SoA interact mutually and positively, the previously demonstrated enhancement of body ownership by interoception may also bolster SoA (22, 23). Second, cardiac interoceptive signals improve the precision of appropriate action selection and control (24, 25), increasing the likelihood that the actual consequences of actions match the desired consequences in the forward models, thus enhancing SoA. Third, a refined ability to integrate interoception and exteroception contributes to SoA, particularly in tasks that rely on exteroceptive feedback, such as the intentional binding task. The beneficial impact of interoception on SoA was significantly associated with interoceptive accuracy that was specifically assessed by the heartbeat discrimination task, reflecting the ability to integrate interoception and exteroception (16). Conversely, abnormalities in interoceptive functioning could have detrimental effects on SoA, particularly in SZ.

There is accumulating evidence for differences and deficits in interoception in SZ, who perform less accurately on heartbeat detection and heartbeat discrimination tasks when compared to non-clinical comparison groups (26–29) or to individuals with other psychiatric diagnoses, including depression and anxiety (21). Several studies also demonstrate a significant association between positive symptoms (hallucinations and delusions) and poor performance accuracy on such tasks (26, 27), although this is not always observed (28). The presence of such interoceptive differences raises the novel proposal that, in schizophrenia, a failure of adaptive integration with interoceptive signals disrupts the SoA, as the expression of a fundamental neuropathological disturbance in the representation and sense of self.

Given the increasing evidence of interoceptive abnormalities in SZ, together with our previous demonstration of a detrimental effect of interoceptive signals on SoA in individuals with poor interoceptive accuracy (16), we hypothesized that there is a differential impact of interoceptive signals on SoA in patients with SZ compared to healthy individuals. More specifically, in the intentional binding task, we predicted a weaker intentional binding effect during systole than diastole in SZ, whereas in healthy individuals, intentional binding would be stronger during systole as has previously been demonstrated (14, 16). In the present study, we tested this hypothesis in an investigation that combines the intentional binding task with heartbeat monitoring. While this cross-sectional study cannot establish causal direction, the assumed pathophysiology associated with this hypothesis is that impaired interoceptive processing could lead to a disrupted SoA, contributing to some of the core symptoms of schizophrenia.

## 2 Methods

### 2.1 Participants

Patients with schizophrenia were recruited from Shimofusa Psychiatric Medical Center.

All patients had schizophrenia, according to Diagnostic and Statistical Manual of Mental Disorder-5 criteria and were clinically stable at the time of testing. Inclusion criteria included (1) 20-65 years old, (2) patients who can perform tasks adequately. Exclusion criteria included (1) patients who are expected to have difficulty in performing tasks due to severe mental symptoms, as determined by clinical judgment, based on patients’ daily life skills and routine medical interviews, (2) patients with physical health complications that affect task performance, such as stroke and brain injury. A comparison group of self-reported non-clinical participants was also recruited as age- and sex-matched healthy controls (HC). All participants were right-handed. To clarify the clinical status of patients, we used the Beck Depression Index-II (BDI) (30), the State-Trait Anxiety Inventory (STAI) (31), and the Positive and Negative Syndrome Scale (PANSS) (32). This study was approved by the Ethics Committee at Shimofusa Psychiatric Medical Center (202112001) and was registered at UMIN (UMIN000047287). All participants gave written informed consent prior to participation. Participants received a 1,000 Japanese Yen gift card as compensation for their participation in the intentional binding task.

### 2.2 The intentional binding task

The intentional binding task was developed to assess the implicit SoA (8). The validity of inferring SoA from this task has been questioned (33) and supported by recent evidence (34). During the task, each participant was required to look at a clock face displayed on a computer screen and to make responses, in accordance with each task condition, by pressing a key with the index finger of the dominant hand. There were 4 conditions and 20 trials in each condition. The order of the conditions was randomized. In the first and second conditions (action-tone condition), the participant was required to make a keypress once during the second rotation of the clock hand. This action caused a tone to be played 250 ms after the keypress. In the first condition, the participant was asked to report the clock hand position at which (when) they made the keypress. In the second condition, the participant was asked to report the clock hand position at which they heard the tone. The third (action-only condition) and fourth (tone-only condition) conditions serve as controls for the first and second conditions. These conditions do not include the causal link between action (button press) and action consequence (tone). In the third condition, the participant was asked to press the button and report the clock hand position at which they pressed the button. No tone occurred in this condition. In the fourth condition, the participant was asked to report the clock hand position at which they heard the tone. No action was required in this condition.

The intentional binding effect, the main outcome of this task, was calculated as the addition of both binding effects on action and tone. The intentional binding effect on action was defined as the difference in reported time between the first and third conditions. Similarly, the intentional binding effect on tone was defined as the difference in reported time between the second and fourth conditions. The (total) intentional binding effect was calculated as the addition of both binding effects. A positive value of the intentional binding effects indicates a shift in the subjective timings of both an action and a tone towards each other when the action is linked to its consequences.

To investigate how cardiac interoceptive signals influence an individual’s SoA, during the performance of this intentional binding task, the participant wore a pulse oximeter (Nonin Xpod 3012LP) on their left index finger, from which pulse times were recorded to identify relevant (systolic and diastolic) periods within the cardiac cycle. This method to identify cardiac cycles was already validated and used in our previous studies (20, 35, 36).

### 2.3 Statistics

**Figure 1:**
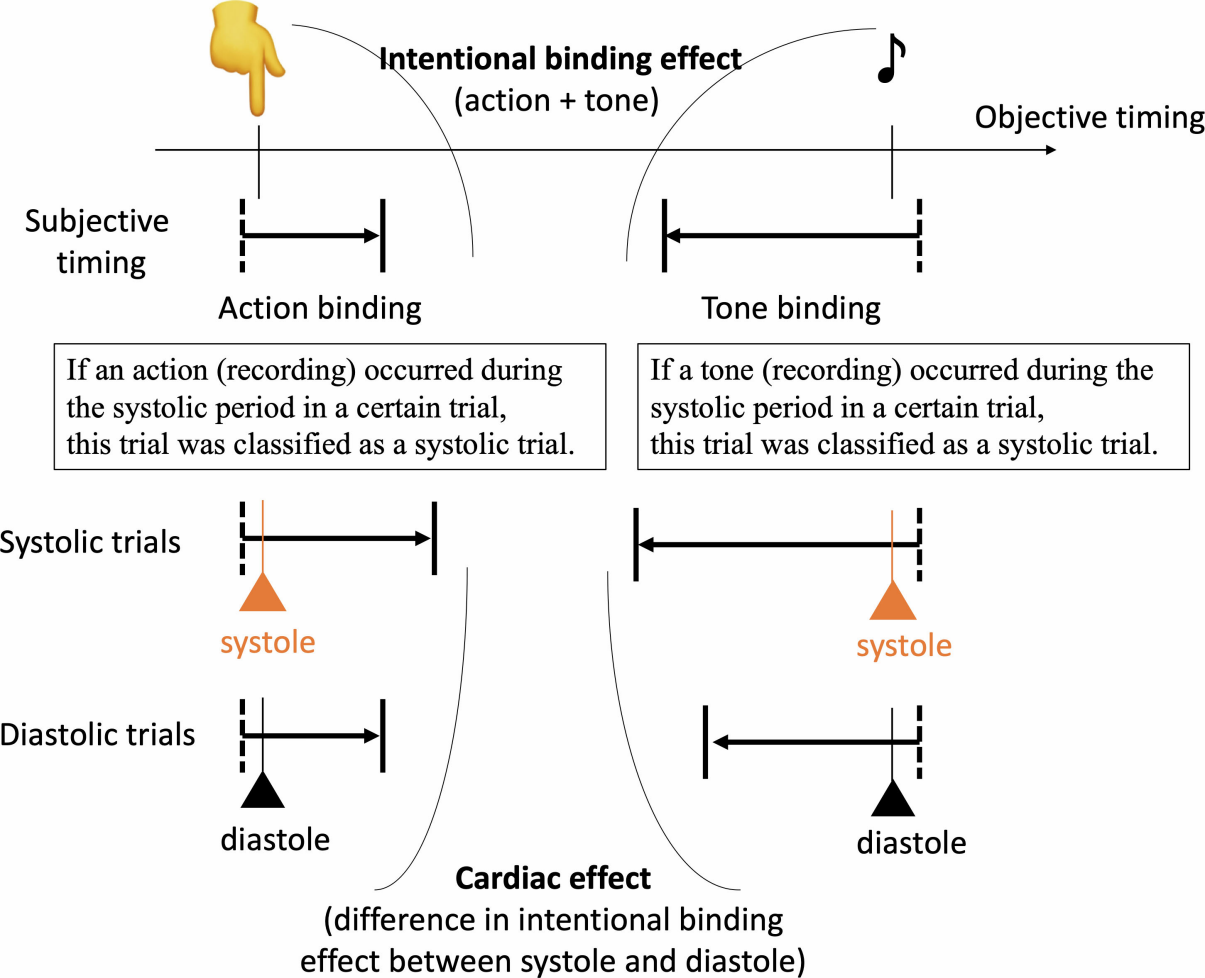
The intentional binding effect was calculated as the discrepancy in self-reported event times between the action-tone condition and the condition involving only action or tone. The cardiac effect was determined by calculating as the difference in the binding effect between systole and diastole, based on objective timings of action or tone. The standardized cardiac effect was then calculated by dividing the cardiac effect by the intentional binding effect and subsequently compared between groups.

Our main analysis was the comparison of the cardiac effect on the intentional binding effect, which was calculated based on the difference in the intentional binding effect between systole and diastole. The definition of whether the keypress and tone in each trial occurred during the systolic or diastolic period was based on a *post hoc* assessment of the pulse recording after the completion of the tasks. As in previous studies, the systolic period was defined as the period of 290 ± 100 ms after R-wave, which was estimated based on their pulse using 250ms as a canonical pulse transit time estimate (20, 24, 36, 37). The diastolic period was defined as the remaining period. Other procedures also followed our previous study (16), see below (**Figure 1**):

Action-tone trials and action-only trials were divided into trials with actions that occurred during systolic or diastolic periods. In a similar manner, action-tone and tone-only trials with reports about tones were divided into trials with tones during the systolic or diastolic period. Each binding effect during systole and diastole was calculated based on each division of trials in accordance with the usual calculation method as mentioned. For example, the binding effect on actions during systole was defined as the subtraction of the reported time of trials with action during the systolic period between the action-tone and action-only conditions. Finally, the cardiac effect on the intentional binding was defined as a subtraction between the binding effects during systole and diastole. A positive value indicates a greater level of the binding effect in systole than in diastole.

Here, as in previous studies, the intentional binding effect itself was greater in SZ than in HC. This difference, attributable to prospective and retrospective components underlying the development of SoA (8, 38), may affect the group comparison of the cardiac effect. To control for this issue, we employed the following formula.


$$\text{Standardised}\;\text{cardiac}\;\text{effect}=\frac{the\;cardiac\;effect\;on\;the\;binding\;effect}{the\;total\;binding\;effect}$$

The standardized cardiac effect refers to the purer cardiac effect, compared to the raw (uncontrolled effect), as it controls for specific, non-cardiac, factors (including prospective and retrospective components) that contribute to the development of SoA.

A general linear model was used to compare the standardized cardiac effect as dependent variables, with groups, age, sex, education, heart rate, BDI, and STAI-State as independent variables. In addition, the population median and the 95% confidence interval (CI) were estimated using the bootstrap method with 1000 iterations. To illustrate our data analysis process, we applied similar models to compare the total intentional binding effect and the unstandardized cardiac effect, respectively. The residuals of our models were normally distributed. Furthermore, to address differences in the total number of systolic trials, which were influenced by individual heart rates, and to account for multicollinearity, we developed another model using the total number of systolic trials instead of heart rate as a sensitivity analysis.

To conduct validated analyses, some participants were excluded from our main analysis, and sensitivity analyses were also conducted to address this issue. While most participants demonstrated the intentional binding effect, two SZ and two HC failed to show this effect, resulting in negative values for their total intentional binding effect, rendering standardization unfeasible. Recognizing the potential risk of heterogeneity in how some individuals conducted the intentional binding task (39), possibly due to inadequate task engagement and/or insufficient effort, we excluded these participants from our analysis. Also, due to the limited overall number of trials and no active synchronization, three SZ and one HC had insufficient systolic trials (only one trial under one of the conditions) to enter into data analysis and were thus excluded from this analysis. Outliers were determined by identifying values beyond the mean minus three times the standard deviation for our main outcome in each group sample, resulting in the exclusion of one SZ. Even after this exclusion, an additional outlier was detected in the remaining sample. To manage the risk of over-elimination and address inclusion concerns, we conducted an additional sensitivity analysis with a more limited sample that excluded the newly identified outlier.

Within the patient group, Spearman correlation analysis, as well as t-tests, were conducted to explore the relationship between the standardized cardiac effect and clinical information. This included scores on the PANSS, the number of hospital admissions, antipsychotic dosage, duration of illness, and patient status (inpatient or outpatient). To address the issue of multiple testing in correlation analyses, the false discovery rate was applied to these analyses. To address our specific research interest and overcome challenges in recruiting severely affected patients, we described the relationship with PANSS-P3 (hallucinations), with P1 (delusions) as a reference, by presenting the values at each level of the PANSS score. Within the diagnostic category of schizophrenia spectrum and other psychotic disorders, schizophrenia is characterized by hallucinations, in contrast to delusional disorder. Hallucination in schizophrenia can be considered a symptom involving a loss of agency (2, 40). A p-value of < 0.05 was considered significant. Statistical analyses were carried out using R (4.3.2).

## 3 Results

### 3.1 Characteristics of participants

Fifty-one SZ and 33 HC undertook the intentional binding task. Three SZ and one HC were excluded due to pulse recording failure. After excluding some participants for the aforementioned reasons for data validation, ultimately, 42 SZ (age 41.9 ± 9.2 years, 22 females) and 29 HC (age 42.7 ± 10.5 years, 15 females) were analyzed. Their characteristics are summarized in **Table 1**. Age and sex were well-matched between groups. SZ participants varied in the recorded number of hospital admissions with a median of 2.0 [2.8] times. The dominant reasons for admission were directly attributable to the expression of hallucinations and delusions, in 91% of cases. for which SZ patients were admitted at a median of 2.0 [2.0] times.

**Table 1:** Characteristics of participants.

	Schizophrenia	Healthy control	p-value
	N = 42	N = 29	
General information			
Age, years, mean ± SD	41.9 ± 9.2	42.7 ± 10.5	0.75
Male, N (%) / Female, N (%)	20 (48) / 22 (52)	14 (48) / 15 (52)	1.00
Education, years, mean ± SD	13.5 ± 2.1	17.0 ± 2.1	< 0.01
BMI, kg/m^2^, mean ± SD	23.6 ± 4.3	24.1 ± 5.2	0.68
HR, bpm, mean ± SD	83.3 ± 17.4	72.9 ± 11.4	< 0.01
BDI, mean ± SD	12.9 ± 10.4	7.6 ± 6.6	0.01
STAI-Trait, mean ± SD	45.5 ± 11.4	41.6 ± 7.8	0.09
STAI-State, mean ± SD	47.5 ± 11.9	40.9 ± 8.7	0.01
Clinical information			
Duration of illness, years, median [IQR]	16.0 [12.5]	–	
Number of hospital admissions, median [IQR]	2.0 [2.8]	–	
Outpatient, N (%) /Inpatient, N (%)	21 (50) / 21 (50)	–	
Drug dosage, CP-equivalent mg, median [IQR]	625.0 [525.0]	–	
PANSS-Positive, median [IQR]	10.0 [7.0]	–	
PANSS-Negative, median [IQR]	13.5 [8.0]	–	
PANSS-General, median [IQR]	28.5 [11.5]	–	
PANSS-Total, median [IQR]	53.0 [25.5]	–	

BMI, body mass index; BDI, beck depression inventory; CP, chlorpromazine; HR, heart rate; IQR, interquartile range; PANSS, positive and negative syndrome scale; SD, standard deviation; STAI, state-trate anxiety inventory.

### 3.2 Comparison of cardiac effect on intentional binding effects

Across those included in the final data analyses, the total number of systolic trials was 26.8 ± 7.2 in the SZ group and 24.3 ± 4.3 in the control group (p = 0.01). For diastolic trials, it was 52.7 ± 7.9 in the SZ group and 55.4 ± 4.5 in the HC (healthy controls) group (p = 0.07). This reflects the group difference in heart rates. The total number of systolic trials and heart rate were significantly correlated (p < 0.01).

**Figure 2:**
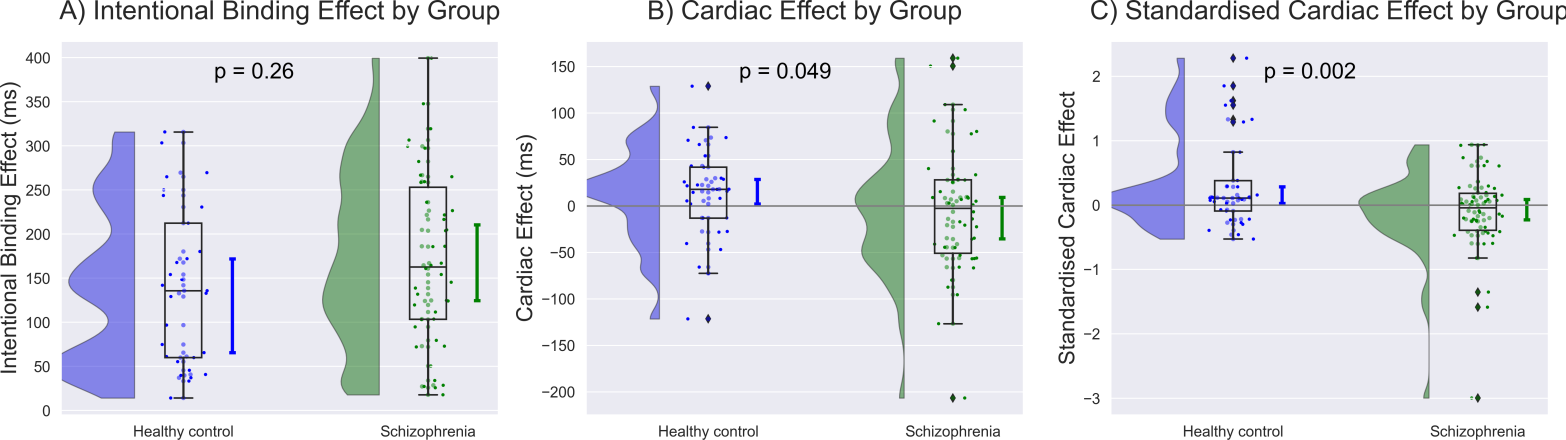
Group differences were demonstrated along with the population median and 95% confidence intervals estimated using the bootstrap method. **(A)** Greater intentional binding effects were observed on average in individuals with schizophrenia (SZ) than in healthy control (HC), although the statistics did not reach a significance. **(B)** The unstandardized value of cardiac effect was significantly smaller in SZ than in HC after controlling for age, sex, education, heart rate, depression, and anxiety. **(C)** The standardized cardiac effect showed a more robust contrast, with a disruption of SoA in SZ and an enhancement in HC. The unit of the standardized cardiac effect is dimensionless.

A comprehensive data report is described in **Table 2**. No significant differences were found in baseline judgment or in the cardiac effect on baseline judgment. The intentional binding effect was median 162.7 [95%CI: 124.7 to 210.6] ms in SZ and 135.8 [65.5 to 172.0] ms, and our model (Akaike information criterion (AIC) = 211) indicated greater intentional binding effects on median in SZ than in HC, although the statistics did not reach a significance (β = 0.31, p = 0.26). The cardiac effect, where a positive value indicates a greater level of the binding effect in systole than in diastole, was -2.6 [-35.2 to 9.3] ms in SZ and 17.8 [2.1 to 28.7] ms in HC, and our model (AIC = 208) indicated a significantly smaller cardiac effect in SZ than in HC (β = -0.63, p = 0.049). The standardized cardiac effect was -0.04 [-0.22 to 0.09] in SZ and 0.11 [0.03 to 0.28] in HC, and our model (AIC = 203) indicated a significantly smaller cardiac effect in SZ than in HC (β = -0.99, p = 0.002) (**Figure 2**, **Table 3**).

**Table 2:** A comprehensive data report.

		Schizophrenia	Healthy control	p-value*
Time about action, ms	Action-tone condition	29.2 ± 104.1	-22.0 ± 60.6	0.20
	Action condition	-13.7 ± 75.7	-46.7 ± 35.3	0.10
	Action binding	42.9 ± 62.9	24.7 ± 50.9	0.80
Time about tone, ms	Action-tone condition	-140.2 ± 125.2	-151.3 ± 81.3	0.68
	Tone condition	-10.8 ± 82.7	-35.5 ± 54.6	0.52
	Tone binding	129.4 ± 86.0	115.8 ± 83.3	0.28
**Intentional binding effect, ms**	Action + Tone binding	172.2 ± 97.9	140.6 ± 90.3	0.26
		162.7 [149.7]	135.8 [152.5]	
Time about action, systole, ms	Action-tone condition	33.6 ± 108.1	-18.0 ± 70.2	0.21
	Action condition	-5.8 ± 104.7	-48.3 ± 39.6	0.06
	Action binding	39.4 ± 69.1	30.3 ± 60.6	0.55
Time about action, diastole, ms	Action-tone condition	25.7 ± 102.3	-23.2 ± 59.0	0.25
	Action condition	-13.6 ± 71.1	-45.1 ± 36.9	0.13
	Action binding	39.3 ± 63.1	21.9 ± 49.4	0.82
Cardiac effect around action, ms (Systole > Diastole)	Action-tone condition	7.9 ± 34.2	5.2 ± 29.5	0.53
	Action condition	7.7 ± 56.9	-3.2 ± 28.2	0.14
	Action binding	0.2 ± 55.0	8.4 ± 37.9	0.31
Time about tone, systole, ms	Action-tone condition	-144.9 ± 131.1	-148.5 ± 83.0	0.73
	Tone condition	-15.8 ± 92.2	-30.1 ± 48.3	0.86
	Tone binding	129.1 ± 103.1	118.4 ± 85.4	0.59
Time about tone, diastole, ms	Action-tone condition	-140.0 ± 126.8	-152.0 ± 83.4	0.59
	Tone condition	-6.3 ± 81.5	-37.1 ± 59.0	0.37
	Tone binding	133.7 ± 84.5	114.9 ± 85.4	0.14
Cardiac effect around tone, ms (Systole > Diastole)	Action-tone condition	-4.8 ± 48.5	3.5 ± 37.8	0.61
	Tone condition	-9.5 ± 38.7	6.9 ± 34.1	0.16
	Tone binding	-4.7 ± 62.0	3.4 ± 47.3	0.18
**Cardiac effect on intentional binding, ms**	Cardiac effect on action + tone binding	-4.5 ± 71.9	11.8 ± 51.9	0.049
		-2.6 [79.2]	17.8 [54.7]	
**Standardised cardiac effect**	Cardiac effect / Intentional binding effect	-0.1 ± 0.7	0.3 ± 0.7	0.002
		-0.04 [0.57]	0.11 [0.47]	

The values are presented as mean ± SD. For our main comparisons, both mean ± SD and median [interquartile range] are shown. * P-values were estimated using general linear models with group, age, sex, education, heart rate, depression, and anxiety as independent variables. The unit of the standardised cardiac effect is dimensionless. Bold values highlight the main results.

**Table 3:** Intentional binding effect, cardiac effect, and standardised cardiac effect.

Variable	Standardised β	Standard error	t-value	p-value
Intentional binding effect				
SZ (vs HC)	0.36	0.32	1.14	0.26
Age	0.17	0.13	1.34	0.19
Female (vs male)	0.19	0.26	0.73	0.47
Education	-0.01	0.16	-0.05	0.96
Heart rate	-0.06	0.13	-0.48	0.64
Depression	0.17	0.18	0.96	0.34
Anxeity	-0.15	0.19	-0.78	0.44
Cardiac efferct on intentional binding effect				
SZ (vs HC)	-0.63	0.31	-2.01	**0.049**
Age	0.07	0.12	0.54	0.59
Female (vs male)	0.17	0.25	0.66	0.51
Education	-0.13	0.16	-0.84	0.40
Heart rate	0.05	0.13	0.36	0.72
Depression	0.14	0.18	0.76	0.45
Anxeity	0.17	0.18	0.90	0.37

Standardised cardiac efferct on intentional binding effect				
SZ (vs HC)	-0.99	0.30	-3.29	**0.002**
Age	-0.11	0.12	-0.91	0.37
Female (vs male)	0.36	0.25	1.45	0.15
Education	-0.24	0.15	-1.59	0.12
Heart rate	-0.11	0.12	-0.92	0.36
Depression	-0.08	0.17	-0.45	0.65
Anxeity	0.24	0.18	1.33	0.19

Bold means significant. SZ, Individuals with schizophrenia; HC, Healthy control. Depression and anxiety were assessed by the beck depression inventory and the state-trate anxiety inventory-state.

### 3.3 Relationship to clinical information

**Figure 3:**
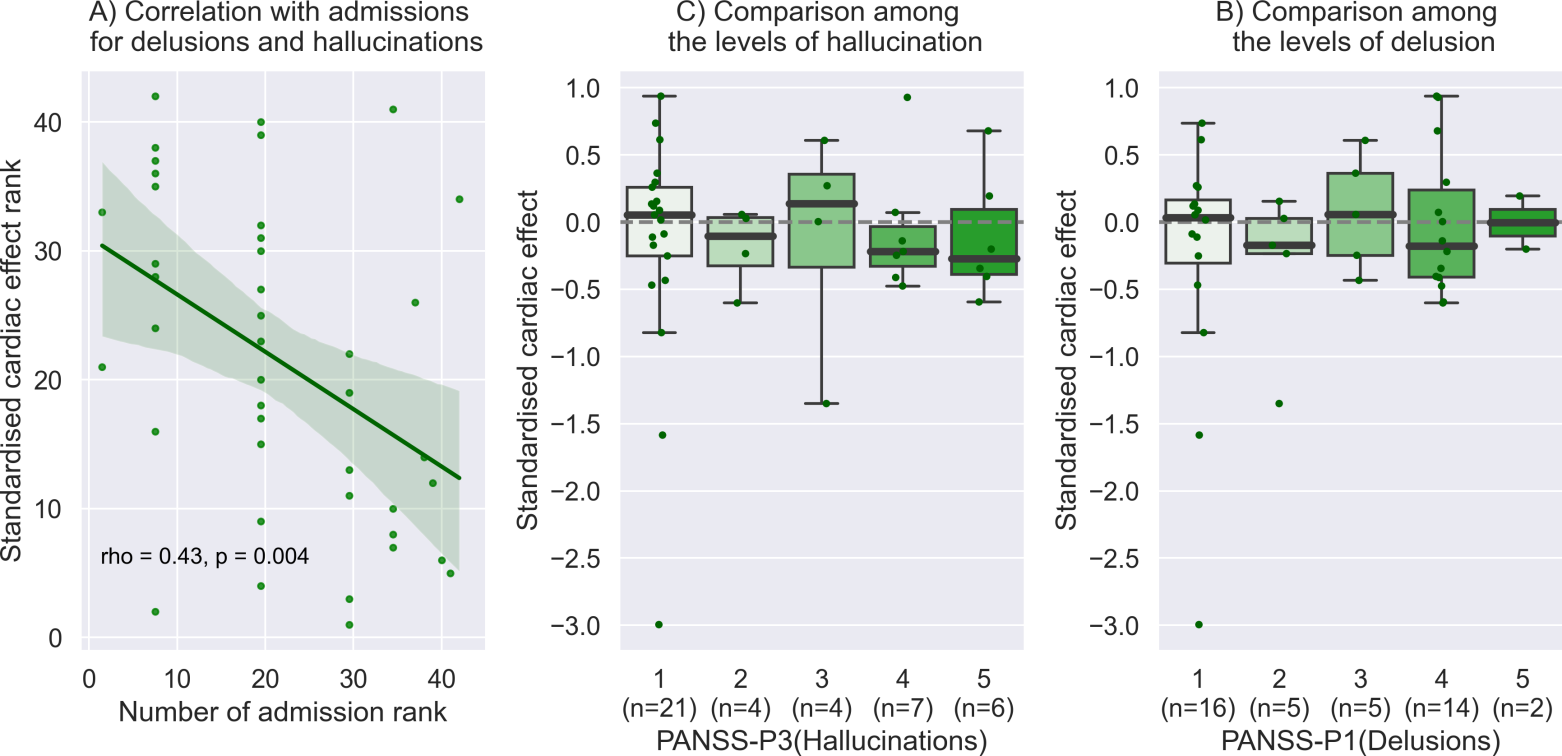
Relationship to clinical information. **(A)** The level of cardiac disturbance of SoA was found to correlate significantly with the number of hospital admissions for delusions and hallucinations. This plot with the Spearman correlation is shown by the rank of each value on the x and y-axis. **(B)** We observed a negative median value of the standardized cardiac effect among patients with severe hallucinations (e.g., median -0.27 [95%CI: -0.50 to 0.43] in the most severe group), with slight gradation depending on the level of hallucinations, although the statistics did not reach a significance. **(C)** No obvious gradation was found with the level of delusions.

The level of cardiac disturbance of SoA (i.e. negative value of standardized cardiac effect on intentional binding) was found to correlated significantly with the number of hospital admissions (rho = -0.39, p = 0.01). When limited to hospital admissions for delusions and hallucinations, the relationship was stronger and more robust (rho = -0.43, p = 0.004) (**Figure 3**). The standardized cardiac effect on intentional binding did not correlate with any other clinical factors, including PANSS-P (rho = -0.06, p = 0.69), -N (rho = 0.07, p = 0.66), -G (rho = -0.0, p = 0.70), antipsychotics dosage (rho = -0.21, p = 0.19), and duration of illness (rho = 0.02, p = 0.89). No difference was found between inpatients and outpatients (t = -0.71, p = 0.49). The significance of the correlation between standardized cardiac effect and hospital admissions for delusions and hallucinations persisted even after applying the false discovery rate correction (corrected p = 0.028). For our specific research interest, we observed a negative median value of the standardized cardiac effect among patients with severe hallucinations (e.g., median -0.27 [95%CI -0.50 to 0.43] in the most severe group), with slight gradation depending on the level of hallucinations, although the statistics did not reach a significance (rho = -0.13, p = 0.42). No gradation was found with the level of delusions (rho = 0.004, p = 0.98).

### 3.4 Sensitivity analysis

In our model using the total number of systolic trials instead of heart rate, no group difference in the intentional binding effect was found (β = 0.34, p = 0.19). The trend level group difference in the unstandardized value of cardiac effect was found (β = -0.61, p = 0.052), but the model of the standardized cardiac effect highlighted the significant group difference (β = -1.03, p = 0.001).

In the model using a more limited sample, no group difference in the intentional binding effect was found (β = 0.51, p = 0.12). The trend-level group difference in the unstandardized value of the cardiac effect was found (β = -0.56, p = 0.08), but the model of the standardized cardiac effect highlighted the significant group difference (β = -0.84, p = 0.009). These sensitivity analyses demonstrated the robustness of our main findings.

## 4 Discussion

This is the first study demonstrating a significant and contrasting difference in the standardized cardiac effect on implicit SoA, as indexed by the intentional binding effect. Specifically, cardiac interoceptive signals, particularly those arising at ventricular systole coincident with each heartbeat, disrupt implicit SoA in SZ, whereas these same signals enhance SoA in HC. The standardization of the cardiac effect by the degree of intentional binding further highlighted this group difference, reinforcing the primacy of the purer cardiac effect by controlling for other factors that influence the development of SoA. Moreover, the group difference in the cardiac effect on implicit SoA cannot be attributed to the differences in heart rate or number of trials, as the significance remained after controlling for these factors. No significant difference in baseline tone or action judgments suggests that our findings pertain more to SoA rather than differences in time perception between the groups. While tone binding appears to contribute more to the cardiac effect than action binding, only total binding achieved significance, indicating the importance of both components. Although all patients were taking antipsychotic medication, the significant association between the magnitude of this disturbance and the number of hospital admissions suggests that this distinct interoceptive effect on SoA contributes to symptom instability. Our findings imply the presence of pathological alterations in brain function supporting interoception and its impact on SoA in schizophrenia: In other words, the interaction between the brain and the heart is compromised, culminating in the disrupted integration of internal bodily states within the representation of self-action.

In this way, the disturbance of the intentional binding effect by interoceptive signals in schizophrenia provides a fundamental pathophysiological mechanism for self-disturbance in schizophrenia that extends and elucidates traditional findings from studies of intentional binding. A pioneering study using this task revealed that the intentional binding effect is stronger in patients with schizophrenia than in non-clinical controls (8). Our finding of greater, but non-significant, intentional binding in SZ aligns with this, although the lack of statistical significance could possibly be due to differences in population and experimental settings. Similarly, explicit measures of SoA are also increased in schizophrenia (5, 41). Nevertheless, a straightforward interpretation of excessive SoA in schizophrenia is somewhat paradoxical when considering the characteristic psychotic symptoms, including loss of agency in actions and thoughts (i.e. delusions of being controlled and verbal hallucinations) (2). To reconcile this paradox, diverse studies, incorporating both prospective and retrospective components, lead to a prevailing theory that SZ patients’ SoA is largely reconstructed retrospectively, based on impaired predictions about action consequences. This contrasts with healthy individuals whose SoA is grounded on predictions (7). Alternatively, the amplification of SoA observed experimentally in patients with schizophrenia may reflect delayed predictions, perhaps attributable to deficits in white matter integrity that affect corollary discharge (6, 42). It should be noted that more recent studies have demonstrated the pathological role of weaker intentional binding in conditions like delusions of being controlled (43) and self-disturbance assessed by semi-structured examinations (44). These findings reflect an inconsistency in this area and highlight the pressing need for additional pathophysiological explanations. Our findings in the current study provide further insight; proposing that abnormalities in central interoceptive processing and representation, in which the interaction between the mind/brain and heart is compromised, underpins the pathological expression of SoA observed in these patients.

As part of normal brain functioning, cardiac interoceptive signals may have a beneficial influence on SoA, potentially in several ways, such as through a mutual and positive interplay with a sense of ownership, improved precision of appropriate action selection and control, and a refined ability to integrate interoception and exteroception (16, 23–25). Conversely, our findings suggest that in schizophrenia, characterized by abnormal interoceptive functioning, cardiac interoceptive signals have a detrimental effect on SoA. In a recently proposed model of the effect of cardiac activity on perception and action (25), the brain-heart interplay involves functional brain changes during the cardiac cycle, optimizing both action (from self to world) and perception (from world to self). This optimization can be conceptualized within frameworks of dynamic gain modulation and precision weighting in the predictive coding theory, that balances interoceptive and exteroceptive signals (20, 25). Here, exteroceptive input is arguably more weighted for perception, while interoceptive input for action. Adaptive functional modulation between action and perception may further support and reinforce self-world boundaries and have an important role in the judgment of self and others (11). SoA is fundamentally aligned to (voluntary) action rather than perception. Previous studies, and our current findings, indicate that SoA is typically strengthened during ventricular systole (14, 16), reflecting enhanced action precision and ownership during this period (23, 25). Thus, inappropriate cardiac modulation, particularly in the context of schizophrenia, may cause inaccurate action control and increase noise during information processing, compromising the experience of SoA. In the intentional binding task, the tone serves as an action consequence and is perceived exteroceptively. Thus, appropriate modulation of both actions and the perception of exteroceptive feedback concerning the consequences facilitate the experience of SoA. It is noteworthy that the strength of an individual’s implicit SoA is shown to be related to their performance accuracy on the heartbeat discrimination task, a test of interoceptive perception that reflects an individual’s ability to integrate interoceptive and exteroceptive information (16). Individuals with schizophrenia perform poorly on the heartbeat discrimination task (21), underscoring the relevance of perturbed coordination between interoception and exteroception to psychotic symptoms.

Furthermore, extending the theoretical role of aberrant or delayed sensory prediction to schizophrenia, aberrant or delayed interoceptive predictions likely contribute to impaired interoceptive functioning in schizophrenia. Heartbeats are involuntary, hence the higher-order generation of visceromotor interoceptive predictions is less direct than action-related predictions, arising from motor commands in a forward model. Nevertheless, efferent visceromotor drive, expressed in states of cardiovascular arousal, is yoked to physical activity and action-ready emotional states that provide both exteroceptive and interoceptive feedback. Here, the predictive coding model can be usefully applied to interoception (45) and offers a framework for self-representation and agency that posits that the brain consistently compares between predicted and actual viscerosensory signals, updating its predictions to minimize discrepancies. We recently observed in an electroencephalography study that individuals with schizophrenia exhibit differences from healthy controls in heartbeat-evoked potentials (a neural signature of cardiac afferent signaling in the brain) that indicate a failure to attenuate interoceptive prediction error (46). Within this predictive coding framework, one can also extend the ‘active inference’ model of prediction error minimization (where action both changes the sensory feedback and improves prediction accuracy) to the handling of interoceptive prediction errors. Here, coherent interaction between the subjective experience of motor action and interoceptive feedback is relevant (12). Thus, aberrant higher order interoceptive prediction may underlie the disrupted SoA observed in schizophrenia. Further studies will help address the question of whether disturbance in selfhood associated with schizophrenia is related to a primary impairment of the afferent interoceptive signals or descending interoceptive predictions (which determine the central representation and impact of interoceptive prediction errors). It is plausible (and likely) that both aspects are compromised. Correspondingly, individuals with schizophrenia exhibit anatomical and functional abnormalities within insular cortex (47), a central neural substrate for interoception, engaged in predictive processing (18, 48, 49).

In addition to SoA, patients with schizophrenia reportedly also demonstrate an opposite cardiac effect on perceptual ratings of emotional stimuli when compared to healthy individuals and individuals with other psychiatric illnesses (35). Here, this perception of fearful facial expressions as more intense and neutral faces as less intense when presented during systole more than diastole, is observed in healthy controls and this effect is further amplified in patients with anxiety. Conversely, individuals with schizophrenia were observed to rate fearful faces as less intense and neutral faces as more intense during systole compared to diastole. This observation suggests an aberrant state where normally attenuated exteroceptive information becomes heightened, aligning with the aberrant salience hypothesis wherein meaningless perception leads to delusional perception in schizophrenia (50). A predictive coding model may equally apply to this. Taken together, perturbed interplay between brain and heart, and abnormal central integration of interoceptive and exteroceptive signals, may lead to disrupted SoA and even delusional perception.

Across the SZ group, we observed a significant association between cardiac disturbances in the SoA and an important proxy measure of severity and clinical instability, i.e. the number of hospital admissions for hallucinations and delusions. This observation indicates a deeper vulnerability arising from a compromised SoA. Although poor adherence to pharmacological treatment is a well-documented contributing factor (51), relapses occur even in patients with good adherence. Thus, the frequency of hospital admissions likely mirrors instability in SoA. Furthermore, cardiac disturbances in the SoA have been particularly observed among patients experiencing severe hallucinations as a state, rather than delusions. Hallucination, especially auditory verbal hallucination, is a symptom specifically associated with schizophrenia compared to delusional disorder, and it can be characterized by a loss of agency (40). While further study is warranted to firmly establish the association among cardiac disturbances, loss of agency, and schizophrenic hallucinations, our findings indicate such potential connections.

The current study has limitations. First, acute patients with severe symptoms could not be included, possibly leading to insufficient power to detect links between the detrimental cardiac effect and positive symptoms. Second, compared to our previous study (16), the number of experimental trials was limited to ensure participating patients were able to perform the task. This also possibly constrained our power to detect some clinical correlations. Third, we used finger pulse to estimate the timing of baroreceptor firing and cardiac phase. This is challenging to do non-invasively even with electrocardiography and may have limiting the precision with which we categorized task events. Fourth, the potential impact of cognitive deficit in SZ cannot be completely denied. However, general cognitive effects (excluding interoception) on intentional binding during systole and diastole may be similar, potentially mitigating the impact on our findings through the subtraction of values between these phases. Fifth, the ‘classic’ intentional binding task cannot dissect the effects of interoception on prospective and retrospective components of the sense of agency. Sixth, given the cross-sectional design of the study, we cannot infer causal directions. Intervention studies involving treatment or manipulation around interoception are required. Some studies on mindfulness, which improves interoception, have shown its beneficial effect on hallucinations in schizophrenia (52, 53). Seventh, the effect of drugs has not been ruled out since all patients were taking antipsychotic medication. While there was no relationship between dosage and cardiac effect in the present study, future research involving drug-naïve patients and pharmacological intervention is required to thoroughly address this issue.

## 5 Conclusion

The observed opposite effect of cardiac interoceptive signals on implicit SoA in individuals with schizophrenia, when compared to non-clinical (healthy) controls, indicates the disrupted interplay between the brain and the heart, and a failure in the coordinated integration of interoceptive and exteroceptive signals within the brain. This pathophysiology likely contributes to the disturbances of coherent self-representation, marking one of the key facets in the development of schizophrenic symptoms. Our findings may have significant implications for the development of novel interventions for schizophrenia.

## Data Availability

The raw data supporting the conclusions of this article will be made available by the authors, without undue reservation.
